# Teacher-Rated Executive Functions, Gender and Relative Age: Independent and Interactive Effects on Observed Fundamental Motor Skills in Kindergarteners

**DOI:** 10.3389/fpsyg.2022.848525

**Published:** 2022-02-22

**Authors:** Elena Escolano-Pérez, Carmen R. Sánchez-López, Maria Luisa Herrero-Nivela

**Affiliations:** ^1^Department of Psychology and Sociology, Faculty of Education, University of Zaragoza, Zaragoza, Spain; ^2^Department of Clinical Psychology, Psychobiology and Methodology, Faculty of Psychology and Speech Therapy, University of La Laguna, San Cristóbal de La Laguna, Spain

**Keywords:** fundamental motor skills, executive functions, gender, relative age, kindergarteners, mixed methods, systematic observation, motor education

## Abstract

Fundamental motor skills (FMS) of children can be affected by different variables, such as executive functions (EF), gender and relative age. However, the effects of these variables on FMS have been scarce studied, especially in early childhood, and show inconsistent results. To clarify these relationships, this study was carried out. Its aim was to analyze whether EF, gender and relative age influenced FMS in 43 Spanish kindergarteners. A multimethod and mixed methods approach was used. Kindergarteners’ teachers completed the Childhood Executive Functioning Inventory to know the children level of EF (working memory and inhibition control). Kindergarteners’ parents complimented *ad hoc* questionnaire reporting the children gender and birth data (to know their relative age). A Nomothetic/Punctual/Multidimensional observational design was used to observe children FMS in their habitual motor sessions at school. Two-way ANOVAs were performed to know the independent and interactive effects of working memory level (lower/higher), inhibition control level (lower/higher), gender (boys/girls) and relative age (according to the birth semester in the year) on FMS. Results showed these variables have independent and interactive effects on some FMS, but not on others. FMS influenced by these variables vary depending what independent variable(s) is/are considered. Therefore, it can be concluded that the influences of teacher-rated EF, gender and relative age on observed FMS in kindergarteners are complex and specific. Results obtained must be taken into to design and implement instructional and intervention strategies, as well as educational and sport policy changes, especially in early childhood, when FMS are more malleable.

## Introduction

Fundamental motor skills (FMS) are organized series of basic movements patterns that involve various body parts to perform a specific act, i.e., they are movements necessary for goal-directed activity (Logan et al., 2018; Lawson et al., 2021). They include three types of skills (Goodway et al., 2019; Bolger et al., 2020): locomotor skills (different movements to transport the body from one location to another such as running or sliding); manipulative or object-control skills (movements to impart force to an object or receive force from an object such as throwing or catching); and stability or balance skills [they are necessary to maintain controlled positions during both static (still) activities, e.g., standing on one leg, and dynamic (moving) activities, such as climbing].

Fundamental motor skills are the foundation or base for future more complex motor skills. In other words, they are the initial building blocks to acquire the more complex specialized skills required in play, games, recreational activities, physical activities and sports for children, adolescents and adults (Hulteen et al., 2018; Logan et al., 2018; Morley et al., 2019; Gu et al., 2021; Lawson et al., 2021). Consequently, failure to develop competency in FMS will make difficult to learn more advanced/specialized forms of these skills (Getchell et al., 2020). In turn, it will decrease the probability of applying motor skills to life-long physical activities, playing sports and becoming an elite athlete (Koch and Krenn, 2021).

The general functional capacity to perform various motor skills is the result of the interaction between: (1) task characteristics, such as its difficulty level depending, for example, if it a rapid coordination of several movements is required; (2) individual genetic and biological factors, such as the height, body weight or the muscle tone, and (3) physical and social environmental conditions, which include, for example, the housing conditions, the opportunity to practice motor activities, the characteristics and variability of these practices or the instructions and feedback received during them (Haywood and Getchell, 2020). Motor skills development shows a protracted maturation trajectory across the life span. It occurs from early childhood (even from the prenatal stage, where the fetus already shows certain reflexes), to early or young adulthood, when peak performance is reached. Later, in middle and late adulthood, they decrease (Goodway et al., 2019; Haywood and Getchell, 2020). This general pattern of development is advocated by different models of motor development such as Gallahue’s Triangulated Hourglass (Gallahue et al., 2012) and Clark and Metcalfe’s Mountain of Motor Development (Clark and Metcalfe, 2002), which are two of the most important models used to help explain motor development. Although these models present some differences in the motor development phases/stages identified within that general model they share, both agree that the development stage of the FMS (extending from ages 2 to 7, approximately) is one of the most important. It builds on the skills learned in the previous period (where the reflexes disappeared and the child’s voluntary behaviors began). At this stage the child begins to establish a fundamental framework for future movements, leading to the establishment of an array of solid movements that enable a quantity and quality of movement skill in later life. Thus, during this stage of FMS development, they go through a defined, observable process from immaturity to proficiency (Goodway et al., 2019; Lawson et al., 2021). The movement choices made later in life (for example, whether an individual ultimately decides to engage in exercise, physical games, sports, or even) will hinge on these FMS. Given that this stage of FMS development coincides with preschool years (Barnett et al., 2019; Wainwright et al., 2019; Wang et al., 2020; Lopes et al., 2021; Shams et al., 2021) and its importance is such that preschool years are considered the golden age for motor development (Wang et al., 2020).

It was previously mentioned that one of the elements that affect motor development and learning is the task characteristics. In this regard, some authors indicate that during the preschool years, given that FMS of children are still in a very intensive stage of development, most of the motor tasks are challenging and new for children (Međedović et al., 2018; Stuhr et al., 2018, 2020). Consequently, to overcome this challenge and novelty that they face, children must implement their executive functions (EF) (Oberer et al., 2017; Stuhr et al., 2018, 2020; Maurer and Roebers, 2019, 2020; McClelland and Cameron, 2019; Brick et al., 2020; De Waelle et al., 2021). EF are higher-order cognitive processes required for goal-directed, adaptive and flexible behavior in novel, demanding, changing or complex situations as well as when learning a new skill, including new motor skills (Zelazo et al., 2016; Maurer and Roebers, 2019; Diamond, 2020). EF are fundamentally required in those situations in which we do not have resolution strategies at our disposal (Zelazo et al., 2016; Diamond, 2020). Their main role is, therefore, supporting action control and facilitating the adaptation of the subject to new situations that continually appear in the context and in which learned responses are not sufficient. Thus, when children are faced with a new and challenging motor task (such as jumping 10 times over the same point with their eyes closed), they need to remember instructions and the goal of the task (e.g., execute 10 jumps on the same point keeping eyes closed); to plan how to do best (e.g., to jump the same pace and strength from the beginning until the end); to implement strategies even in case of distraction (e.g., not to be influenced by other children); or inhibit impulses (e.g., open eyes or stop jumping). All these processes are part of EF and are necessary to solve the new and challenging motor task. Therefore, EF influence motor performance. Consequently, children with a strong skill set of EF can better perform the motor tasks (Houwen et al., 2019). There are studies that go further and indicate that infant EF can even predict the athletic success of an athlete, being children with higher levels of EF the ones with the strongest chance to become elite athletes (Koch and Krenn, 2021). Thus, there are several studies that show that individual differences in children’s EF are concurrently and predictively related to execution in motor and sport tasks (Krenn et al., 2018; Ishihara et al., 2019).

Executive functions, like FMS, show a prolonged development throughout the life cycle. EF begin to develop very early (7–12 months). Between 2 and 6 years of age they show significant and rapid development that will continue later, albeit at a slower rate, until early adulthood (around 20 years), when the maximum level of executive development is reached (Diamond, 2013; Zelazo and Carlson, 2020; Zelazo et al., 2021). Thus, as in the development of FMS, the preschool years represent one of the most critical period for EF development.

At this stage of life there are significant improvements in numerous processes related to EF, in parallel with important changes of the prefrontal cortex, both at the structural and functional levels (as such wide pruning of synaptic connections; maturation of subcortical prefrontal myelination; change in the activity pattern of relevant areas; etc.) as well as in other brain regions and connections (such as parietal, temporal, or hippocampal areas) (Smith et al., 2017; Zelazo and Carlson, 2020; Zelazo et al., 2021). These rapid changes that occur in EF during the preschool years make it difficult to determine the structure and organization of EF at this stage of life (Scionti et al., 2020), which justifies the existence of different models in the literature about the processes that make up children’s EF (Stalnacke et al., 2019). While there is consensus about the multidomain structure of EF in adulthood, the structure of EF in childhood is still an open question (Metaferia et al., 2021). Different studies have found empirical evidence both in favor of a one-dimensional model and of multidimensional models formed by two factors/functions or even three, which nature varies according to studies (Miyake and Friedman, 2012; Diamond, 2013; Stalnacke et al., 2019; Hartung et al., 2020; Scionti et al., 2020). Despite these discrepancies, a certain consensus currently begins to exist which indicates that EF are a unidimensional construct in early childhood but multidimensional one in late middle childhood and beyond. However, the time when EF structure changes from an undifferentiated one-factor to a multidimensional model is still up for debate. Recent studies indicate that at 5–6 years (ages of the participants in this study) the best model to summarize and explain the structure and organization of EF is a two-factor structure comprising working memory and inhibition control, considered as diverse but united components (Monette et al., 2015; Simanowski and Krajewski, 2019; Stalnacke et al., 2019; Scionti et al., 2020). Working memory refers to the capacity to retain information in mind while mentally manipulating it to perform tasks (Baddeley, 2012). Inhibitory control implies the voluntary control of goal-irrelevant stimuli, cognitions, and behavioral responses (Nigg, 2000; Diamond, 2013, 2020). It restrains the impulse to react, providing space for focus and decision. Inhibitory control enables an individual to persist in problem solving and attain future goals through managing competing/conflicting stimuli (maintaining focus on a relevant cue while ignoring an irrelevant cue), and suppressing automatic or not appropriate responses to the task at hand (Tiego et al., 2018).

In short, and according to various authors (McClelland and Cameron, 2019; Michel et al., 2020), EF are cognitive prerequisites to successfully perform FMS tasks during preschooler period, which implies that individual differences in children’s EF are concurrently related to running on FMS tasks. Thus, the term motor, in childhood, implies cognition. However, they have traditionally been considered and studied as two separate developmental domains, so that studies about the influence of EF on FMS in childhood ages are scarce and inconsistent. Hence our interest in the subject and the conduct of this study in order to help eliminate this gap.

On the other hand, the literature indicates that, in addition to the EF, other relevant variables that also seem to influence the execution of FMS tasks are gender and relative age, i.e., age difference between individuals of the same group, or what is the same, difference in days between people born in the same calendar year (Navarro-Patón et al., 2021). However, the studies that analyze these sociodemographic variables and preschool FMS are scarce and show disparate results. That is why it was considered interesting to include both variables (gender and relative age) in this study.

Regarding the influence of gender on FMS, more and more studies show that during the preschool years there are differences in the level of certain FMS that students acquire based on their gender. At this early stage of life, these differences do not seem to be due to maturational issues but rather to differences in the socialization of boys and girls and/or in the opportunity that each of them has to carry out some motor activities or others. Thus, the social context could be of great importance when it comes to practicing different motor skills and, consequently, achieving a higher degree of performance in one or the other (Bolger et al., 2020). Several studies indicate that boys and girls show different levels of competence in some FMS but not in others, in which there are no gender differences (Kokštejn et al., 2017; Matarma et al., 2020). However, there is no total consensus when determining in which FMS these differences are shown, or not, and if these differences are in favor of one gender or another. Thus, with regard to locomotor skills, the discrepancies between studies are large, with evidence both toward the absence of differences (Bakhtiar, 2014; Foulkes et al., 2015; Barnett et al., 2016; Bolger et al., 2018, 2020; Honrubia-Montesinos et al., 2021) and in favor of them. Among the latter studies, some find that girls outperform boys (Bolger et al., 2018, 2020; Wang et al., 2020), and others show opposite results (Robinson, 2010; Pahlevanian and Ahmadizadeh, 2014). Regarding control object skills, most of the works show that boys outperform girls (Foulkes et al., 2015; Barnett et al., 2016; Venetsanou and Kambas, 2016; Kokštejn et al., 2017; Bolger et al., 2018, 2020; Honrubia-Montesinos et al., 2021; Mecías-Calvo et al., 2021), although there are also studies that do not find differences between the two genders (LeGear et al., 2012; Bakhtiar, 2014). About balance skills, there is again disparity of results: some indicate that girls’ performance on the balance tasks is significantly better than boys (Pahlevanian and Ahmadizadeh, 2014; Venetsanou and Kambas, 2016; Kokštejn et al., 2017; Mecías-Calvo et al., 2021) and others show no differences between the two genders (Singh et al., 2015; Barnett et al., 2016). This disparity of results about the existence or not of differences between genders in FMS requires further research. It is necessary to know if there are weaknesses and strengths of girls and boys in different FMS in order to determine educational priorities or retrieve different motor skills weaknesses in each gender.

Concerning relative age and its influence on FMS, there are also no conclusive results on the existence, or not, of significant differences in the FMS level of preschool children born during the same calendar year. In the educational system (context where this study was developed), and as in the rest of systems (sports, social.), a series of criteria is established based on which to group the collectives (students). This pretends to minimize significant differences between the subjects that make up this group and, therefore, to be able to adjust the difficulty of the proposed tasks to the average maturational level, avoiding differences in their result or training. In the Spanish educational system (where this study was carried out) the criterion for grouping children is the year of their birth. All children born in the same calendar year belong to the same grade. However, although as already mentioned, this grouping mode tries to minimize the differences between the children of the same grade, being reduced to one chronological year, this difference is enough for the so-called relative age effect (RAE) to appear: that is, the effects (advantages or disadvantages) that occur as a function of the difference in the month of birth with children belonging to the same group, or what is the same, the consequences attributed to the chronological age difference between individuals belonging to the same group. These effects or consequences in the area in question, the FMS, would be specified in that children who were born shortly after the cut-off date (beginning of the year), that is, the oldest, would show higher levels of FMS than the smallest (born later in the year). However, recent studies indicate that this is not always the case: RAE seems not to be evident in all FMS. However, there is no consensus about which FMS evidence RAE and which do not. Regarding control object skills, some studies show RAE on these skills (Imbernón-Giménez et al., 2020; Navarro-Patón et al., 2021) and others do not (Imamoglu and Ziyagil, 2017; Mecías-Calvo et al., 2021). A similar situation is found for balance skills: some studies reveal RAE (Mecías-Calvo et al., 2021) and others do not (Imbernón-Giménez et al., 2020; Navarro-Patón et al., 2021). Regarding RAE on locomotor skills, although the results are disparate between the studies once more, more specific results have been found in that only some of the locomotor skills (not all) have RAE. However, other authors disagree when finding that none of these skills have RAE (Imbernón-Giménez et al., 2020). This variety of results requires further investigation. Determine whether or not there is RAE on the FMS level of preschoolers, and if there are, in which ones, it is essential since it implies knowing if certain preschoolers (the youngest) are at a disadvantage with respect to their older companions. This disadvantageous situation could have effects not only in the short term but also in the medium and long term in children. Younger children, in addition to presenting lower levels of FMS, have a greater probability of being less physically active and abandoning sports more, in addition to a lower probability of being subsequently selected to enter sports training programs, being full members of good sports teams and become sports stars (Pérez-González et al., 2021). Therefore, if there is RAE on FMS, the school or sport staff will have to create equal opportunities for all children, taking into account that equal opportunities does not mean that everyone must learn the same thing at the same time, but rather implies that making sure that all students have the best possible opportunities to grow into their full potential. This will require an individualization of teaching, adapting it to the needs of the students, and therefore, it will mean addressing the heterogeneity of the students and the educational responses that are offered to them (OECD, 2018; UNESCO, 2018).

Taking into account all the above, and in order to contribute to the different gaps already mentioned, this study was carried out. Its aim was to analyze independent and interactive effects of executive functions, gender and age relative on FMS in preschoolers (exactly, in 5–6 years old kindergarten children).

Based on the existing literature, the following hypotheses were proposed:

H_1_: Differences will be found in FMS based on the level of executive functions: children with higher level of EF will have higher FMS level than children with lower level of EF.

H_2_: Some differences will be found in FMS based on gender: boys will show higher levels than girls in some FMS, while in other FMS girls will show higher levels. In other FMS there will be no differences between boys and girls.

H_3_: Some differences will be found in FMS based on relative age: older kindergarteners (i.e., born in the first half of the year) will show higher scores in some FMS than their younger peers (born in the second half of the year). On the other hand, in other FMS there will be no differences between the kindergarteners born in different semesters.

Given our lack of knowledge of studies that had jointly addressed all the variables of interest in our study, no hypothesis was raised regarding the interaction effects of the variables.

Determining the nature of the possible effects between these variables may offer valuable information to teachers, movement specialists and other professionals who work with children during the critical developmental phase of FMS – in addition to researchers and politicians to design and implement instructional and intervention strategies, curriculum development, and policy changes that contribute to the development of physically active people and even future sports stars.

## Materials and Methods

### Methodology and Design

We used a multimethod (use of multiple methodologies to address different goals within a research project) and mixed methods (integration of qualitative and quantitative components to address the same aim) perspective (Anguera et al., 2018a). The multimethod perspective consisted of selective methodology to know information referring to the sample’s inclusion/exclusion criteria (questionnaire and a standardized battery were used), the sociodemographic variables of gender and relative age (questionnaire was used), and to assess EF (questionnaire), in addition to observational methodology to assess kindergarteners’ motor skills in their habitual motor skills sessions in their school. Given that observational methodology itself is nowadays considered a mixed methods (because it achieves total integration between qualitative and quantitative elements), it can be affirmed that the study was also carried out from a mixed methods perspective (Anguera et al., 2018a,2020a,2020b). In the observational methodology the integration between qualitative and quantitative elements is achieved thanks to the succession of QUAL-QUAN-QUAL macro-stages. The first QUAL stage implies to build an *ad hoc* observation instrument and to apply it to code the behaviors that are interesting for the study, considering the natural setting in which they occur. The QUAN stage involves to test the observational data quality and to carry out the analyses using quantitative techniques to respond to the study aims. In the following and last stage (QUAL stage), the quantitative results obtained are qualitatively interpreted. In this way, full integration between qualitative and quantitative elements is achieved (Anguera et al., 2020b).

Observational methodology plays a crucial role in this study since it is the idoneous methodology for studying the behavior of young children, and, more specifically, those that occurs in natural contexts such as the school (Bakeman and Gottman, 1987; Anguera, 2001; Escolano-Pérez et al., 2017, 2020; Cohen et al., 2018; Palaiologou, 2019; Anguera et al., 2020a,b). The observational design followed, according to the classification established by Anguera et al. (2018b), was Nomothetic/Punctual/Multidimensional. It was “Nomothetic” because different participants were individually observed, more exactly, 43 kindergarteners were studied; “Punctual” because for each kindergartener, we collected data referring to each motor ludic task in an observation session; and “Multidimensional” because various response levels referred to distinct aspects of FMS were observed (They compose the observation instrument).

The observation was based on scientific criteria, active (given the aim was determinate), non-participatory (the observers did not interact with the kindergarteners) and direct (the level of perceptibility of the behaviors was complete). It was performed by direct observation of recorded film (Anguera et al., 2018b). The recommendations of the Guidelines for Reporting Evaluations based on Observational Methodology (GREOM) (Portell et al., 2015) and the Methodological Quality Checklist for Studies based on Observational Methodology (MQCOM) (Chacón-Moscoso et al., 2019) were followed.

### Participants

The study participants were recruited using non-probabilistic convenience sampling approach. They were 43 Spanish kindergarteners aged 5 to 6 years (*M* = 68.6 months; *SD* = 3.59; range = 69–74 months). Being kindergartener in Spain (country where the study was carried out) implies being a student in the last year of the second cycle of Pre-school Education or Early Childhood Education and Care, which corresponds to International Standard Classification of Education (ISCED) level 020- and consequently, be 5–6 years old. Early Childhood Education and Care in Spain is a not mandatory educational stage, but currently 98% of 5-year-olds are enrolled in it (Spanish Ministry of Education and Professional Training, 2020). Of all participants, 15 (34.88%) were boys and 28 (65.12%) were girls. All had a medium-high socioeconomic level and were students of the same kindergarten (although they attended 2 different groups/classes). It was a publicly funded private school located in the center of a city in Northern Spain.

Inclusion/exclusion criteria were the following, in agreement with other similar studies such as Van Der Veer et al. (2020). As inclusion criteria were applied: (1) having completed the entire second cycle of Early Childhood Education and Care in this same center (or what is the same, being a student of this school since the age of 3); (2) normal or corrected to normal hearing and vision; and (3) adequate IQ according to their chronological age. Exclusion criteria were: (1) birth weight < 2,000 g and/or gestational age < 36 weeks or significant pre, peri-, or postnatal events; (2) signals of a medical condition, genetic, psychiatric or neurological disorder, or physical disability.

The study was part of a broader research project endorsed by the Research Unit of the university to which the first author belongs. School staff approval and written consent from the parents was obtained. The participants were treated according to the international ethical principles indicated in Declaration of Helsinki.

### Instruments

#### Instruments Used for Selective Methodology

In order to obtain information about inclusion criteria 1 (having completed the entire second cycle of Early Childhood Education and Care in the same center where the study was conducted) and 2 (normal or corrected hearing and vision), in addition to the exclusion criteria 1 (birth weight < 2,000 g and/or gestational age < 36 weeks or significant pre, peri-, or postnatal events) and 2 (signals of a medical condition, genetic, psychiatric or neurological disorder, or physical disability), and gender and date of birth (to be able to calculate relative age), *ad hoc* questionnaire to be completed by the kindergarteners’ parents was used.

To become acquainted with information related to inclusion criterion 3 (adequate IQ according to the chronological age) the Battery of Differential and General Abilities I (BADyG-I; Yuste and Yuste, 2001) was used. It is a reliable and very useful Spanish standardized battery to assess IQ in students between 4 and 6 years of age. It is composed of six tests that allow knowing the Verbal IQ (evaluated through three tests: Numerical Quantitative Concepts; Information and Graphic Vocabulary) and the Non-verbal IQ (evaluated with three other tests: Non-verbal mental ability; Reasoning with Figures and Logic puzzle). Adding both (Verbal and Non-verbal IQ) also gives the General IQ. In each of the items that make up the battery (a total of 108 items, 18 in each of the 6 tests), the evaluator reads the statement of the item aloud. The child must mark the correct answer with a cross, selecting it from among five images that appear in the answer booklet. For example, in the Information test, in each item the child must select the drawing that meets the definition given by the adult. In the Reasoning with Figures test, each item consists of selecting the draw that differ the most from the others.

To assess kindergarteners’ EF, the Spanish-language version of the Childhood Executive Functioning Inventory (CHEXI) was used (Thorell and Nyberg, 2008). It is a very useful questionnaire that allows to detect executive deficits in children aged 4–12 (Thorell and Catale, 2014; Acero-Ferrero et al., 2017; Camerota et al., 2018; Pérez-Pereira et al., 2020). CHEXI is composed by 24 items/statements divided in two subscales: working memory (13 items, e.g., “Has difficulty remembering lengthy instructions”); and inhibition control (11 items, e.g., “Has difficulty holding back his/her activity despite being told to do so”). It can be responded by children’s teachers or parents in a 5-point Likert scale indicating the extent to which each item describes the child’s habitual behavior, where 1 = absolutely uncertain; 2 = not true; 3 = partially true; 4 = true; 5 = very true. Higher scores indicate greater executive deficits, and therefore, worse executive functioning, while lower scores indicate good executive functioning. CHEXI has been translated into different languages showing good psychometric properties (Thorell et al., 2010; Thorell and Catale, 2014; Catale et al., 2015). The questionnaire is free available for download in several different languages from www.chexi.se. The Spanish-language version (used in this study) can be downloaded from https://chexi.se/onewebmedia/CHEXI_Spanish.pdf.

#### Instruments Used for Observational Methodology

##### Observation Instrument

The observation instrument used combined a field format and category systems, according the multidimensionality of our observational design. It was composed by 21 criteria and 71 categories which fulfilling the requirements of exhaustiveness and mutual exclusivity. They were adapted from an observation instrument built *ad hoc* for a previous study (Escolano-Pérez et al., 2020). This instrument allowed observing different elements of children behavior referring to FMS. The observation instrument used is available in Supplementary Material.

##### Recording Instruments

A video digital camera was used to record kindergarteners’ motor sessions. The Lince software program (Gabin et al., 2012) was employed to code the behaviors referred to FMS in accordance with the observation instrument. It can be free downloaded from http://www.observesport.com.

#### Data Analysis Software

IBM SPSS Statistics 25.0 software package (IBM Corp, 2017) was used to analyze the data.

### Procedure

The research team held an informational meeting with the school management team to explain the research and request its collaboration. Once this was obtained, another meeting was held with the teachers and, later, another with the parents. Informed consent and the questionnaire prepared *ad hoc* (to know the information referred to the inclusion criteria 1 and 2; the two exclusion criteria; the gender and date of birth of their child) were given to the parents. In case of authorizing the participation of their child in the study, the parents had to deliver both completed documents (consent and questionnaire) to their child’s teacher 10 days later.

To the kindergarteners who met the inclusion criteria 1 and 2 and did not present the exclusion criteria, the research team administered the BADyG-I (Yuste and Yuste, 2001) to check if they met the inclusion criterion 3 (adequate IQ according to their chronological age). Its application was collective in each group/class. For each group, it was applied in two sessions of approximately 30 minutes, on non-consecutive days, following the instructions of the battery manual both in this aspect and in others (distribution and order of administration of the tests in each session). BADyG-I was always administered by the same researcher, with three more researchers who provided support ensuring that all participants answered each item in its corresponding place in the answer booklet. To correct the BADyG-I, the computer-automated procedure offered by the battery itself was used.

To assess kindergarteners’ EF, teachers completed the CHEXI (Thorell and Nyberg, 2008) in reference to each of their students. Each teacher had 15 days to do so, being free to do so at the most suitable time for her (either during school hours or outside). After this time, each teacher returned the completed questionnaires to the research team.

To observe the kindergarteners’ FMS, different ludic motor activities/tasks were designed. In order to make them age-appropriate tasks, the Spanish Early Childhood Education and Care curriculum (Spanish Ministry of Education and Science, 2007) and theorical and empirical studies about motor development in kindergarten students were taken into account (Lincolnshire Community Health Services NHS Trust, 2018; De Waal, 2019; Goodway et al., 2019). In the design of the motor tasks, the physical and temporal characteristics of the natural context where the research was carried out were also considered (that is, the dimensions of the school’s motor skills room and the materials available in it, as well as the schedule and duration of habitual motor sessions). Despite the fact that different authors defend that most of the motor tasks are new and challenging for children, and therefore imply the implementation of EF, to ensure that the designed tasks meet these characteristics (unfamiliar, challenging and demanding), they were explained and shared with the children’s teachers. Thus, it was ensured that the type of designed tasks and motor patterns required in them had not been worked on in their usual motor sessions: that is, participants did not have experience with this kind of movement (at least in their usual school motor sessions). In addition, given that according to the literature, motor practice and repetitions of a task reduce its novelty, making it easier and more familiar, and decreasing the involvement of EF in its execution (Međedović et al., 2018); and (2) given that certain motor patterns can be learned very quickly, even with only two trials (Maurer and Roebers, 2020), it was decided that the child only had a single trial in each task. In this way, we tried to make sure again that the tasks was new and challenging for children and therefore, they implied recruitment of EF (Spedden et al., 2017; Stuhr et al., 2018, 2020).

From all designed tasks, eight motor tasks were selected to be used in this study. The execution of each task, according to the literature (Goodway et al., 2019; Lawson et al., 2021), fundamentally reflected one of the domains of FMS: locomotor, object control, static balance and dynamic balance (already explained in section “Introduction”). So, each domain of FMS was evaluated through the execution of two tasks. Table 1 describes these tasks and indicates which FMS was fundamentally required in the execution of each of them.

**TABLE 1:** Motor tasks and fundamental motor skills (FMS) required in the execution of each of them.

Motor tasks	Description motor tasks	FMS	
Hopping with one leg	Jump on the limp by moving (without stopping) on each of the sides (1 m long) of a square drawn on the ground. Two consecutive sides must jump on the same leg; and the other two, on the other leg.	Locomotor skills	
Long jump	Jump with your feet together as far as possible, starting from a given point, and land without touching the ground with your hands.		
Throwing a ball	Throw a tennis ball with one hand so that it passes through a hoop placed at a distance of 1.5 m.	Object control skills	
Catching a ball	Catching a tennis ball thrown by an adult from a distance of 1.5 m.		
Squatting	Squat on the balls of the feet, keeping the feet 30 cm apart, the body bent, the arms extended horizontally to the sides and the eyes closed. Maintain this position for as long as possible.	Static	Balance skills
Standing on one leg	Stand on one leg with your eyes closed, for as long as possible. It is done first with one leg and then with the other.		
Walking heel-to-toe	Walk on each of the sides (1 m long) of a square drawn on the ground, placing the heel of the foot to be supported next to the tip of the foot already supported, without leaving space between them.	Dynamic	
Vertical jumps	Jump vertically 10 times in a row, always landing at the same starting point, and always keeping your eyes straight ahead.		

The motor tasks were performed in the usual motor sessions that were part of the children’s ordinary school schedule, respecting, as already mentioned, their distribution in the calendar and their duration. These and other organizational aspects of the school and curricula determined (1) that for each class group, four motor sessions were necessary on alternate days, and (2) that the distribution of tasks was the following: first session, hopping with one leg and squatting tasks; second session, throwing a ball and walking heel-to-toe tasks; third session, standing on one leg and long jump tasks; fourth session, vertical jumps and catching a ball tasks. Each teacher, explained and executed each task once before it was completed by their students, thus using their conventional motor teaching strategies. The performance of each participant in each of the tasks was recorded with a video camera.

Subsequently, an expert observer, both in kindergarten FMS and observational methodology coded the recorded sessions. To carry out it, video recordings were imported into the Lince software and were coded using the observation instrument.

The quality control of the observational data was carried out both qualitatively and quantitatively. Regarding qualitative perspective, consensual agreement was used to get an agreement between two observers (observer 1 and another observer also expert in kindergarten FMS and observational methodology). It was used in three sessions for each activity (therefore, a total of 24 sessions). Subsequently, data quality control was performed using quantitative perspective, calculating both intra-observer and inter-observer reliability. To calculate the intra-observer reliability, observer 1 coded all the sessions a second time, comparing them with the records that she had previously carried out. To calculate inter-observer reliability, observer 2 also coded all sessions. Her records were compared with those that observer 1 made for the first time.

### Data Analysis

To calculate the quality of the observational data, a classical perspective was used in which the correlations were assessed both between the categories and between the criteria of the observation instrument coded by observer 1 in her two records (intra-observer reliability), as well as the correlations between the categories and the criteria coded in the first record of observer 1 and those coded by observer 2 (inter-observer reliability), through the correlation coefficients of Pearson, Tau-b of Kendall and Spearman. In addition, Cohen’s Kappa coefficient was used, which refers to the concept of association. In all the cases, intra-observer agreement was ≥ 0.90; and inter-observer agreement was ≥ 0.70. Thereby, the quality of the data was guaranteed.

In order to carry out the pertinent analyzes that would allow responding to the study aim, it was previously necessary to prepare the data as follows: (1) Based on the scores obtained by the participants in each of the two EF evaluated (working memory and inhibition control), two groups were created. That is, based on the scores obtained in working memory, two groups were established: one corresponding to those participants who showed a lower level of working memory (therefore, higher scores obtained in the work memory scale of the CHEXI, since as already mentioned, this questionnaire assesses difficulties in EF) and another group corresponding to those who showed a higher level of working memory (lower scores on this scale of the CHEXI). To establish the cut-off point between both groups, the group median was considered as a criterion, thus following the same grouping procedure as that used in other studies in the educational field (Diaz-Bilello and Briggs, 2014; Honrubia-Montesinos et al., 2021). The same procedure was carried out with the scores referring to inhibition control, that is, two groups were established (lower and higher level of inhibition control) using the median of the group as a criterion. (2) Considering the gender of kindergarteners, two groups were created: boys and girls. (3) Taking into account the date of birth of the participants, and more specifically, based on the semester of the year in which they were born, thus, using the same grouping criterion that other studies (Kasuga et al., 2012; Imbernón-Giménez et al., 2020; Martínez-Moreno et al., 2020) two groups were established: group 1 = children born in the second semester of the year, i.e., from July 1 to December 31. Thereby, they were the youngest participants (range age: 63–68 months); group 2 = children born in the first semester of the year, i.e., from January 1 to June 30. They were the oldest participants (range age: 69–74 months). (4) Regarding the observational data referred to FMS, it was necessary to transform them as follows. For each participant and task, each category observed during the execution of the task was transformed into a score based on the degree of suitability that the category implied for the execution of the task, according to the literature on the subject (Payne and Isaacs, 2017; Goodway et al., 2019; Haywood and Getchell, 2020). For each participant and task, the scores referring to the categories observed in said task were added, thus obtaining eight scores for each participant (one per task). Subsequently, for each participant, the scores of the two tasks in which the same FMS were involved (indicated in Table 1) were added, thus obtaining four new scores referring to: locomotor skills, object control skills, static balance and dynamic balance. Adding these scores, each participant obtained a final score referred to total FMS. In this way, a total of 13 scores were obtained per participant.

Descriptive statistics (group means and standard deviations) were calculated. It was found that in the models used the data fulfilled the assumptions of normality of the distribution, homoscedasticity and independence of the observations.

Two-way ANOVAs were performed. Levels (lower/higher level) in working memory and inhibition control, gender (boys/girls) and relative age (youngest/oldest participants) were used as independent variables, and scores referring to FMS skills as dependent variables. All *p*-Values < 0.05 (two-tailed) were considered statistically significant. The effect size was calculated through Cohen’s *d* (Cohen, 1988).

## Results

The results obtained indicate that there are significant differences in some FMS depending on the level of working memory of the participants, but not in others. Specifically, there are differences in the scores referring to the following FMS: long jump [*F*(1,34) = 8.593, *p* = 0.006; *P* = 0.810; *d* = 1.28; CI: 95%: 1.12–1.45]; total locomotor skills [*F*(1,34) = 8.880, *p* = 0.005; *P* = 0.825; *d* = 1.27; CI: 95%: 1.1–1.43] and total FMS [*F*(1,34) = 6.912, *p* = 0.013; *P* = 0.724; *d* = 1.11; CI: 95%: 1.05–1.17]. Children with higher working memory obtained higher scores on this FMS (Table 2).

**TABLE 2:** Means (M) and standard deviations (SD) obtained in the fundamental motor skills (FMS) in which there are significant differences based on the working memory level.

FMS score	Lower working memory		Higher working memory	
	*M*	*SD*	*M*	*SD*
Long jump	30.38	16.97	51.08	15.15
Total locomotor skills	52.08	17.46	73.92	16.87
Total FMS	255.15	20.90	283.80	29.70

Taking into consideration the level of inhibition control of the participants (lower/higher level), significant differences are found in the following FMS scores: vertical jumps [*F*(1,34) = 10.389, *p* = 0.003; *P* = 0.879, *d* = 1.75; CI: 95%: 1.53–1.97]; total dynamic balance [*F*(1,34) = 13.107, *p* = 0.001; *P* = 0.940, *d* = 0.76; CI: 95%: 0.66–0.85]; total balance [*F*(1,34) = 9.192, *p* = 0.005; *P* = 0.838; *d* = 0.29; CI: 95%: 0.17–0.40] and total FMS [*F*(1,34) = 7.456, *p* = 0.01; *P* = 0.756; *d* = 0.93; CI: 95%: 0.66–1.19]. In all of them, the group of children with higher inhibition control obtained higher scores (Table 3).

**TABLE 3:** Means (M) and standard deviations (SD) obtained in the fundamental motor skills (FMS) in which there are significant differences based on the inhibition control level.

FMS score	Lower inhibition control		Higher inhibition control	
	*M*	*SD*	*M*	*SD*
Vertical jumps	47.13	16.69	71.20	9.84
Total dynamic balance	82.44	17.15	95.55	17.15
Total balance	145.65	16.85	150.28	14.22
Total FMS	259.50	21.32	284.55	31.48

There are significant gender differences in squatting [*F*(1,34) = 6.679, *p* = 0.014; *P* = 0.709; *d* = 0.83; CI: 95%: 0.72–0.94] and total static balance [*F*(1,34) = 6.846, *p* = 0.013; *P* = 0.720, *d* = 0.78; CI: 95%: 0.68–0.88]. Girls outperformed boys in both FMS scores (Table 4).

**TABLE 4:** Means (M) and standard deviations (SD) obtained in the fundamental motor skills (FMS) in which there are significant gender differences.

FMS Score	Boys		Girls	
	*M*	*SD*	*M*	*SD*
Squatting	26.25	10.13	34.15	8.66
Total static balance	52.08	11.29	60.46	9.92

Results show significant differences based on relative age in vertical jumps [*F*(1,34) = 9.108, *p* = 0.005; *P* = 0.834; *d* = 0.64; CI: 95%: –0.018–0.146] and total dynamic balance [*F*(1,34) = 10.981, *p* = 0.002; *P* = 0.896; *d* = 0.77; CI: 95%: 0.72–0.86]. In both cases, the oldest participants obtained higher scores than the youngest ones (Table 5).

**TABLE 5:** Means (M) and standard deviations (SD) obtained in the fundamental motor skills (FMS) in which there are significant relative age differences.

FMS Score	Younger participants		Older participants	
	*M*	*SD*	*M*	*SD*
Vertical jumps	59.45	18.87	69.28	10.82
Total dynamic balance	85.15	15.81	95.44	10.30

**FIGURE 1:**
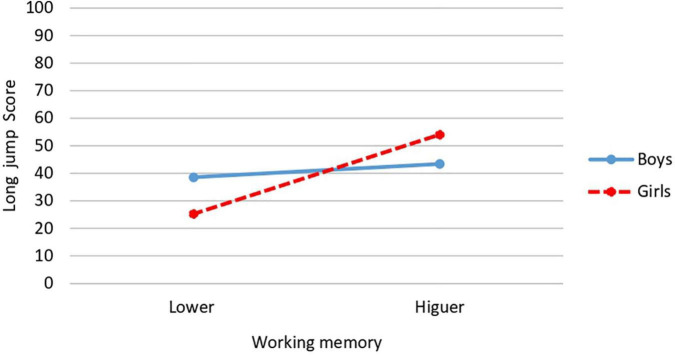
Interaction effect of working memory and gender on long jump.

**FIGURE 2:**
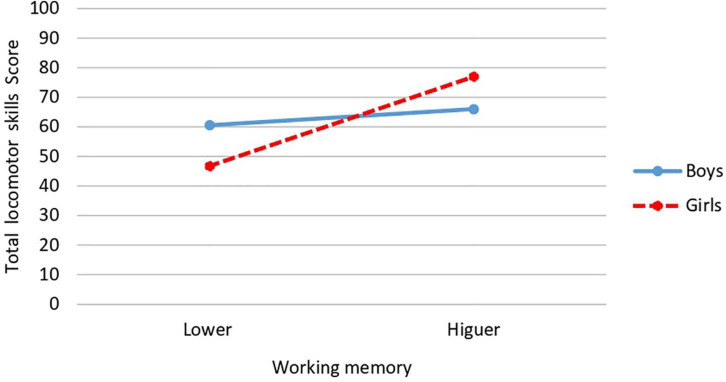
Interaction effect of working memory and gender on total locomotor skills.

Regarding the working memory × gender interaction, significant differences are obtained in long jump [*F*(1,34) = 4.34, *p* = 0.045; *P* = 0.521; *d* = 2.12; CI: 95%: 1.86–2.41], obtaining the highest score in girls with higher working memory (*M* = 54.06; *SD* = 13.05) and the lowest score in girls with a lower level of working memory (*M* = 25.25; *SD* = 14.06) (Figure 1). Significant differences are also obtained in total locomotor skills [*F*(1,34) = 4.315, *p* = 0.045; *P* = 0.523, *d* = 2.01; CI: 95%: 1.73–2.28], where, as in the case above, the highest and lowest mean was obtained in girls. The highest in those who presented higher working memory (*M* = 77; *SD* = 15.27) and the lowest in those who presented lower working memory (*M* = 46.75; *SD* = 13.84) (Figure 2).

**FIGURE 3:**
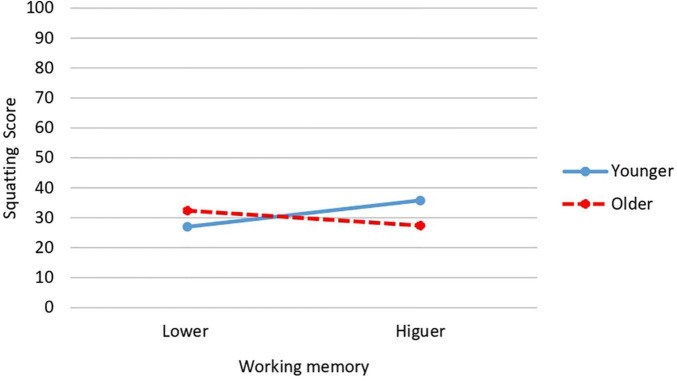
Interaction effect of working memory and relative age on squatting.

The working memory × relative age interaction produces significant differences in squatting [*F*(1,34) = 4.445; *p* = 0.042; *P* = 0.553; *d* = 1.03; CI: 95%: 0.71–1.35]. The highest mean was obtained by the youngest participants with higher working memory (*M* = 35.80; *SD* = 8.64) and the lower mean by children also younger but with lower working memory (*M* = 27; *SD* = 8.38) (Figure 3).

**FIGURE 4:**
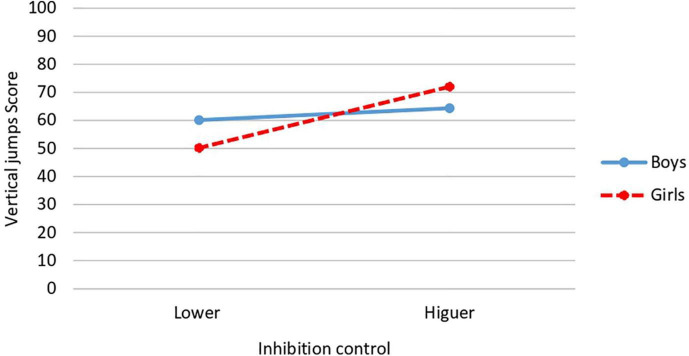
Interaction effect of inhibition control and gender on vertical jumps.

**FIGURE 5:**
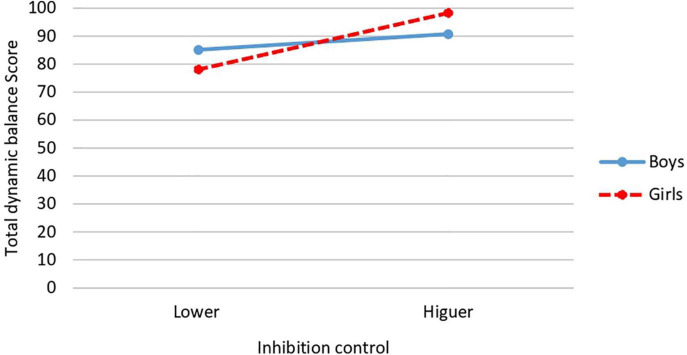
Interaction effect of inhibition control and gender on total dynamic balance.

About inhibition control × gender interaction, significant differences are obtained in the FMS of vertical jumps [*F*(1,34) = 96.18, *p* = 0.004; *P* = 0.854, *d* = 3.1; CI: 95%: 2.5–3.7] and total dynamic balance [*F*(1,34) = 8.370, *p* = 0.005; *P* = 0.834; *d* = 1.57; CI: 95%: 1.27–1.87]. In the case of vertical jumps, the highest mean corresponded to girls with higher inhibition control (*M* = 72.05; *SD* = 4.06) while the lowest mean was obtained by girls with lower inhibition control (*M* = 50.22; *SD* = 17.39) (Figure 4). In the case of total dynamic balance, and as in the previous case, the highest mean occurred in girls with higher inhibition control (*M* = 98.29; *SD* = 4.40) and the lowest mean in girls with lower inhibition control (*M* = 78.08; *SD* = 17.64) (Figure 5).

**FIGURE 6:**
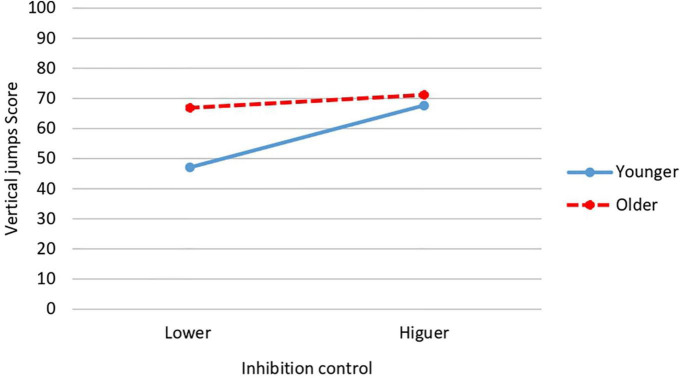
Interaction effect of inhibition control and relative age on vertical jumps.

**FIGURE 7:**
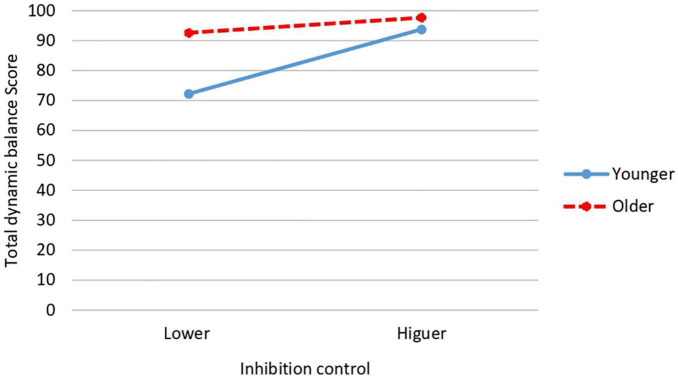
Interaction effect of inhibition control and relative age on total dynamic balance.

Inhibition control × relative age interaction shows significant differences also in vertical jumps [*F*(1,34) = 9.108, *p* = 0.005; *P* = 0.834; *d* = 1.75; CI: 95%: 1.63–1.87] and in total dynamic balance [*F*(1,34) = 10.981, *p* = 0.002; *P* = 0.896; *d* = 1.95; CI: 95%: 1.79–2.11]. In vertical jumps, the highest mean is obtained by the oldest participants with higher inhibition control (*M* = 71.20; *SD* = 9.84) while the lowest scores are obtained by the youngest participants with lower inhibition control (*M* = 47.13; *SD* = 16.69) (Figure 6). In total dynamic balance, exactly the same occurs: the highest mean is obtained by the oldest participants with higher inhibition control (*M* = 97.70; *SD* = 9.09) while the lowest score is obtained by those of younger age and lower inhibition control (*M* = 72.25; *SD* = 16.06) (Figure 7).

Gender × relative age interaction did not show significant differences in any of the FMS studied.

## Discussion

The aim of this study was to analyze whether there were independent and interactive effects of executive functions (attending to the level of working memory and inhibition control), gender and age relative on kindergarteners’ FMS. The results obtained indicate that these variables have independent and interactive effects on some kindergarteners’ FMS, but not on others. The FMS that are affected by these variables vary depending on which variable or variables (working memory, inhibition control, gender or relative age) are involved. Therefore, it can be concluded that only certain FMS are specifically influenced by some of these variables.

Regarding the power of the test, which was carried out because the sample size was small, it was adequate (>0.80) or close (>0.70) in most of the analyses. However, in some cases (working memory × relative age, and working memory × gender) the power of the test decreased. Nonetheless, Cohen’s *d* was good in all the cases, except in one of them (inhibition control effect on total balance). To corroborate the results in these cases, the sample size should be increased (as we indicate later, when study limitations are mentioned).

More specifically, and in relation to the first hypothesis postulated (kindergarteners with higher level of EF will have higher FMS level than their peers with lower level of EF), the results found only allow us to partially corroborate this hypothesis, given that higher levels of EF are only associated with higher levels of certain FMS but not all. Furthermore, depending on whether it is a higher level of working memory or inhibition control, the FMS in which higher scores are obtained vary. Thus, it has been found that participants with higher working memory obtained higher FMS scores in long jump (referring to locomotor skills), total locomotor skills and total FMS, but not in the remaining FMS. Therefore, neither the control objects skills nor the balance skills are influenced by the level of working memory. In fact, not even the level of working memory affects all locomotion tasks, since the performance in hopping with one leg (which, like long jump, implies locomotor skills) does not vary depending on the level of working memory. This denotes the specificity of the relationships between the working memory level and the FMS. Similar results are found in regard to the inhibition control levels in that only some FMS are affected by this variable. Participants who showed higher inhibition control obtained higher scores in vertical jumps (referred to dynamic balance skills), total dynamic balance, total balance and total FMS. Thus, only some skills related to balance, and especially some related to dynamic balance such as vertical jumps (but not all of them such as walking heel-to-toe), in addition to the other total scores mentioned, are influenced by the level of inhibition control. None of the locomotor and object control skills assessed in this study are affected by the level of inhibition control. Again, these results reflect the specificity of the relationships between inhibition control and FMS.

The specificity of all these results are difficult to explain because of a lack of previous studies focusing on this topic. It is a very complex and open issue for further research. Nevertheless, some studies have tried to address this question taking into account different separable stages of acquisition that can be identified during the motor sequence learning. The different EF components would have a fundamental role in one or other stages of motor learning. For example, working memory would be involved in updating motor control after an error in order to improve de execution for subsequent trials (Seidler et al., 2012). Since in our study the participants only had one trial in each task, this argument would help explain why working memory hardly affected FMS in our study. However, it does not allow understanding why long jump, total locomotor skills and total FMS are affected by working memory. Another issue that could explain our results is the different pattern of development shown by working memory and inhibition control. Although a central time window for the development of EF is the first 5 years of life, each EF component may follow a somewhat different developmental trajectory (Ackerman and Friedman-Krauss, 2017). Working memory shows a somewhat linear improvement from preschool stage through adolescence, while inhibition control presents a rapid increase during the preschool stage followed by a moderate improvement rate thereafter (Best and Miller, 2010). It could be that many of the preschoolers that make up our sample had not yet developed a sufficient level of working memory to used it, i.e., the steady development of working memory would be still underway. However, the rapid improvement in inhibition control might already have occurred in them, and consequently they might have used it. In that regard, it seems reasonable that the interplay between EF and motor skills is different and mutable as long as the development of each EF are occurring (Stuhr et al., 2020). More research is needed to obtain evidence to support these arguments. In conclusion, our findings support the idea that the performance of the different FMS tasks differ in the degree to which the different EFs are required. In other words, the role that each EF plays for the performance of the different FMS tasks is specific, so that several types of FMS depend on the effectiveness of different EFs. Or in other words, motor performance in some tasks, but not in others, is not an independent process of the level of efficiency of different EF. All this shows that the relationships between EF and FMS are specific and very complex.

These results are in line with those obtained by other authors (Stuhr et al., 2018; Ludyga et al., 2019) who did not find behavioral evidence to support a global-to-global relationship between higher-order cognitive abilities (EF) and motor skills in typically developing children. Instead, support founded for some distinct and specific associations between both aspects in preschoolers (Wassenberg et al., 2005; van der Fels et al., 2015; Houwen et al., 2017; Stuhr et al., 2018). However, the few studies that have investigated the relationships between EF and motor performance in preschool children show inconsistent results about what these specific relationships are (van der Fels et al., 2015; Stuhr et al., 2018). Livesey et al. (2006), like us, found that working memory was related to locomotor skills. Regarding inhibition control, and unlike our results, there is evidence in literature on its influence on the three types of FMS: locomotor skills (Roebers and Kauer, 2009), object control skills (Livesey et al., 2006) and balance skills (Rigoli et al., 2012; Stuhr et al., 2018), this last aspect is the only one that coincides with our results. On the other hand, considering the relationships between inhibition control and total motor skills, our results are consistent with those of Livesey et al. (2006), who also found associations between inhibition and overall motor performance in typically developing 5- to 6-year-olds. However, making a direct comparison between the studies is complex and should be done with caution, since the studies present differences at a theoretical level, methodological perspective used, tasks used, etc. (Leonard and Hill, 2015; Ruddock et al., 2016; Houwen et al., 2017). In this regard, several studies suggest that motor competence could vary even between tasks considered similar since the relationships between EF and motor skills in preschool children are task-specific (Roebers and Kauer, 2009; Michel et al., 2011; Stöckel and Hughes, 2016; Van Der Veer et al., 2020). This helps to explain some of the most striking results found in our study, in that not all tasks related to locomotion nor all those related to dynamic balance were affected by EF level. According to various authors (Roebers and Kauer, 2009; Michel et al., 2011; van der Fels et al., 2015; Stöckel and Hughes, 2016; Houwen et al., 2017), we consider that more research is required that includes a more exhaustive variety of FMS tasks to gain a deeper understanding of the complex and specific relationships between EF and FMS as well as the specialized processes that underlie these relationships. It is a very complex issue given, in addition, these specific EF-FMS relationships differ depending upon the age and ability level of the participants under investigation (Stuhr et al., 2020).

Numerous neuroimaging, clinical and developmental studied have been carried out trying to clarify the interaction between EF-FMS. They have shown that cognitive and motor performance share some common underlying processes, i.e., there is an overlap in neural networks that are important for both EF and FMS since: (1) some regions important to cognitive and motor performance are co-activated both during cognitive and motor tasks (Diamond, 2000; Leisman et al., 2016); (2) cognitive and motor problems often co-occur in children with neurodevelopmental disorders (Higashionna et al., 2017), and (3) prefrontal cortex (area traditionally considered the neural base of EF) and the cerebellum (important in the motor aspect) show a common developmental timetable (Ludyga et al., 2019). This evidence is in line with the notion that cognitive and motor performances are inter-related, but does not elucidate the specific specialized processes underlying the different EF and motor skills relationships. In this regard, although much remains to be known, recent studies show that cerebellar cortex exhibits fine-scale functional specialization similar to the degree of specialization observed in cerebral cortex (Brissenden and Somers, 2019; Stein, 2021). So, different cortico-cerebellar networks may play highly specific functional roles in a broad range of cognitive processes. To determine what are these different cortico-cerebellar networks activated based on different aspects (age of the individual, his/her experience, complexity of the movements, characteristics of the task, etc.) is still an open question to the researchers. There are still scarce number of studies in this field and no conclusive explanations or conclusions have been found so far (van der Fels et al., 2019). Consequently, as already mentioned, future research is needed.

The results obtained have shown that having certain high levels of EF help to better perform certain FMS tasks, since EF would help children to better control their body, retain movement sequences and form mental representation, focus on what is relevant and ignore other distracting stimuli (Diamond, 2020; Stuhr et al., 2020). This, and especially the specificity of the relationships between EF and FMS, must be taken into account when designing interventions. Thus, it is expected that interventions targeting specific EF influence specific motor skills, yet not all motor skills. According to this assumption, professionals should choose interventions carefully by selecting those that target the specific skills of interest.

Although our study was carried out in the school context, its results also have implications in other areas, such as the field of physical and sports activity, and especially, the preparation and selection of future sports talents. Preschoolers with certain higher EF may be more likely to reach the highest levels of certain FMS and consequently, could reach the highest performance levels in certain sports, whereas preschoolers with lower EF may be more likely to reach lower levels of certain FMS and consequently, they would be more likely to drop out the motor and physical-sports activities or not reach elite status (Ishihara et al., 2019; Koch and Krenn, 2021). This means that the level of certain childhood EF establish if a child has the capacity to reach top levels in some sports and allows distinguishing between potential elite and non-elite athletes in said sports (Krenn et al., 2018; Holfelder et al., 2020). Therefore, this implies that EF constitutes a variable to take into account for the identification of sports talents, in addition to physical and technical performance usually considered (Ishihara et al., 2019; Scharfen and Memmert, 2019; Sakamoto et al., 2021).

This study also tried to analyze whether there were gender differences on FMS. H_2_ hypothesized that there would be differences in certain FMS between boys and girls, outperforming one gender or another in different FMS, but not in others. The results obtained allow us to partially corroborate the hypothesis insofar as there are gender differences in some FMS scores (squatting and total static balance) but not in others (all the remaining FMS scores). However, unlike what was postulated, in those FMS where there are gender differences, it was always the girls who obtained higher scores. These results are hardly directly comparable with those obtained in other studies given the differences between studies in age and other characteristics of the participants, tasks and evaluation tools used, etc. Despite this, it can be said that our results are congruent with those of some previous works, but not with those of others. Thus, the absence of gender differences in locomotor skills found in our study is in line with the results of previous studies (Bakhtiar, 2014; Foulkes et al., 2015; Barnett et al., 2016; Bolger et al., 2018, 2020; Honrubia-Montesinos et al., 2021). On the contrary, other studies found both differences in favor of girls (Bolger et al., 2018, 2020; Wang et al., 2020) and in favor of boys (Robinson, 2010; Pahlevanian and Ahmadizadeh, 2014). Non-gender differences on control objects skills obtained in our study are also coincident with the results of other studies (LeGear et al., 2012; Bakhtiar, 2014), but they do not match with numerous works who defend that boys outperform girls (Foulkes et al., 2015; Yang et al., 2015; Barnett et al., 2016; Venetsanou and Kambas, 2016; Kokštejn et al., 2017; Bolger et al., 2018, 2020; Honrubia-Montesinos et al., 2021; Mecías-Calvo et al., 2021). Regarding balance skills, comparing our results with those of other studies becomes even more complex since few studies consider static and dynamic balance separately. Some studies, even using tasks referring to both types of balance skills, finally offer only a sum total score of both (Kourtessis et al., 2008; Engel-Yeger et al., 2010). These studies also present conflicting results. Some indicate that even at the preschool age, girls have higher scores than boys on balance tasks (Fjørtoft, 2000; Lejarraga et al., 2002; Lam et al., 2003; Sigmundsson and Rostoft, 2003). However, other studies show no significant differences between preschool boys and girls (Du Toit and Pienaar, 2002; Venetsanou, 2007; Kourtessis et al., 2008; Waelvelde et al., 2008; Singh et al., 2015). Among the few works that differentiate the two types of balance, the following results are detected. Several studies, like ours, show gender differences in static balance (Demura et al., 1994; Demura, 1995; Livesey et al., 2007). It is striking that Livesey et al. (2007) found these gender differences using a task that has also been used in this study (standing on one leg), but in which we have not found gender differences (instead, we have found them in the other task referred to static balance, squatting, and in total static balance). These discrepancies between studies, despite having used the same task with preschoolers, may be due to the different parameters used in the assessment of their performance. While Livesey et al. (2007) only considered the time that children were kept on the limp —product oriented evaluation—, we have attended to numerous criteria —both product and process oriented—. (These criteria can be consulted in the observation instrument, available on the Supplementary Material). Regarding dynamic balance, some research (Livesey et al., 2007; Aoki et al., 2011, 2015; Kasuga et al., 2012; Latorre-Román et al., 2021), and in line with the results of this study, show that there are no differences between genders in preschoolers. The non-gender differences on total FMS showed by our results are also consistent with those of other studies (Hardy et al., 2010; Kordi et al., 2012). However, these results disagree with other investigations that found that boys demonstrated higher levels of total FMS compared to girls (Barnett et al., 2009; Cohen et al., 2015; Charlesworth, 2016). Once again, the variety of results found in the literature highlights the need for further research to learn more about the specific relationships between gender and motor skills.

However, in general and given the few preschoolers’ FMS scores in which gender differences have been detected in our study (only squatting and total static balance scores), we can conclude that our results are in line with the general assumption defended by some authors who indicate that for most of the motor skills, there are no consistent gender differences before adolescence (Handelsman et al., 2018). At this stage of life, adolescence, a significant concentration of testosterone is produced in boys that contributes to creating bigger and stronger bones, as well as greater muscle mass and strength. This produces a significant improvement in their motor skills, generating differences with respect to girls (Xu et al., 2021). However, in stages prior to adolescence, the absence of important physical differences for motor skills would explain why boys and girls, in most FMS tasks, show similar performance. Nonetheless, other authors, according to a socio-ecological approach (Bronfenbrenner and Morris, 2006; Mehtälä et al., 2014) and Dynamical Systems Theory (Thelen and Smith, 1996; Newell and Liu, 2014) defend that social and environmental aspects cannot be forgotten, since not only maturation but also practice helps to improve motor skills. In this way, the existence or absence of differences in FMS proficiency between genders during early childhood could be ascribed to a complex interaction of biological, environmental, and sociocultural factors, such as the physical environment and the influence of family, peers, and teachers (Kokštejn et al., 2017; Newell, 2020). In this sense, it has been found that even in “modern” countries, the types of opportunities offered to children to practice FMS and engage in structured FMS programs vary according to gender, which can affect the level and type of FMS competence demonstrated by each of them (Spessato et al., 2013; Venetsanou and Kambas, 2016). Thus, the higher dynamic balance score obtained by the girls participating in our study could be due, at least in part, to the greater practice of the girls of activities that promote this type of skills (such as dancing and gymnastics) than the boys (Chatzopoulos et al., 2018). In other types of activities and experiences (such as running, volleyball or handball) that essentially enhance other motor skills (locomotor and object control skills), currently the gender difference in their practice is less than in dancing or gymnastics (Nielsen et al., 2011). This could help explain the absence of significant gender differences in our study in this type of motor skills (locomotor and object control skills).

All these aspects indicate that, when offering opportunities to practice FMS skills, gender stereotypes are maintained more with boys than with girls (Rodríguez and Miraflores, 2018). This is consistent with studies carried out in other areas (for example, professional vocation) in which it is also detected that traditional gender role beliefs are more strongly endorsed in boys than in girls (Dicke et al., 2019; Hentschel et al., 2019; Spinner et al., 2021). Thus, there is a change in gender stereotypes traditionally assumed by girls, assuming traits and activities considered typically masculine, but there is no change in the opposite direction (Gustafsson et al., 2019). It could be that the policy of equality in sport and, in general, the policies of empowerment of women, in which Spain has been working for a few years, are bearing fruit. However, in view of our results, we consider it relevant to also pay attention to the experiences that are being offered to boys from the earliest ages. Children’s gender stereotype knowledge rapidly increases from 3 years of age through direct and indirect experience, and observation of their social worlds. At 5–6 years, adherence to gender stereotypes seems to peak. In this age children are highly rigid in their thinking about and observance of gender stereotypes, so that gender stereotypes can act as a barrier to children’s practice motor or physical activities deemed appropriate for the other gender (Spinner et al., 2021). Given that these years coincide with the Early Childhood Education and Care stage, and practically all children attend it, it acquires a very relevant role in curbing gender stereotypes and working from co-education. However, if education practitioners are not aware of the essential role they play in this matter, they can also spread gender stereotypes. They have their own implicit beliefs and subconscious understandings of gender and this is transferred to the different expectations and different ways of interacting with their students depending on their gender (Culhane and Bazeley, 2019; King et al., 2021). This is key to the way in which children learn about gender. Therefore, as a first step to stop gender stereotypes, it is necessary that education practitioners (but also other professionals who work with minors) are aware of how their beliefs and practices may be favoring gender stereotypes. On the basis of this knowledge, they will be able to practice a “gender flexible teaching” (Culhane and Bazeley, 2019). For all this, including mandatory training on this subject in the training plans for teachers and other professionals would be a useful strategy. Of course, creating parenting schools to help families to “gender flexible parenting” would also be necessary, since they also have their own implicit and subconscious belief about gender that they transmit in their parenting patterns to their children. Collaborations between practitioners and researchers would also need to be facilitated from the policymakers in order to design effective interventions against gender stereotypes, few to date (Spinner et al., 2021).

H_3_ hypothesized that some differences would be found in FMS based on relative age. More exactly, H_3_ stated that older kindergarteners (born in the first semester of the year) would show higher scores in some FMS than their younger peers (born in the second semester of the year); but in other FMS there would be no relative age differences. This hypothesis has been corroborated since the oldest kindergarteners only obtained significantly higher scores than their younger companions in vertical jumps (a task related to dynamic balance) and total dynamic balance. There are no significant differences in the other FMS scores. These results corroborate those of other authors as they did not find RAE on locomotor skills (Imbernón-Giménez et al., 2020) or in control object skills (Imamoglu and Ziyagil, 2017; Mecías-Calvo et al., 2021). However, they are contrary to those studies that reported RAE on some locomotor skills (Imamoglu and Ziyagil, 2017) as well as in control object skills (Imbernón-Giménez et al., 2020; Navarro-Patón et al., 2021). Regarding balance skills, some studies, in line with our results, show RAE on these skills (Mecías-Calvo et al., 2021) and others, on the contrary, do not (Imbernón-Giménez et al., 2020; Navarro-Patón et al., 2021). However, these studies do not differentiate between static and dynamic balance. More research is needed to gain more insight on this topic. In addition, as previously indicated, several discrepant aspects between studies (methodological differences, tasks and measures used to assess functioning in each domain, specific ages and other characteristics of the preschoolers that made up samples, etc.) may be contributing to the disparity of results.

In summary, and despite the fact that some studies show RAE on FMS, in our study most of these skills were not affected by RAE (specifically, only two FMS were affected). Consequently, we can conclude that there was limited RAE on the FMS of the studied participants. These results are very similar to those found by Imamoglu and Ziyagil (2017), who state that, in generally, RAE at the age of 5–6 years did not influence the FMS.

Many studies have tried to justify the existence of RAE on motor skills by assuming that older children were biologically more mature and, thus, physically and functionally superior in comparison to their relatively younger peers (Baker et al., 2010). Other authors, according to a socio-ecological approach and Dynamical Systems Theory (Bolger et al., 2020), and therefore, highlighting the role that the child’s environment also plays in motor development, have also highlighted the increased opportunities of older children for motor practices, experiences, and feedback, which helped to refine their motor skills. However, in relation to this last aspect, opportunities for motor practices, there are studies that indicate that it is not so important the “when” is born (that is, the relative age) but the “where” (Côté et al., 2006); understanding the “where” in a broad sense. Thus, this would include the place of early development and its contextual factors such as the experiences offered in it, among which are the motor support and training programs (Campbell et al., 2019). In this sense, there are studies that indicate that in Western societies FMS are essentially promoted in school, where the child spends more hours a day (Lopes et al., 2021). In this regard, let us remember that one of the inclusion criteria for the participants in this study was to have completed the entire second cycle of Early Childhood Education and Care at the same school they attended when the study was carried out. Given that school is the context where children spend the most hours, and it was the same for all participants, it could be thought that their motor experiences were similar, which would help to explain that only two FMS had RAE in our study. Furthermore, it should be noted that regarding the possible motor experiences of our participants outside of school, the literature indicates the following. In “small” or “medium” size communities (between 1,000 and 499,999 inhabitants), the effects of RAE among children are lower than in large communities (more than 500,000 inhabitants), since in the former the motor experiences are more accessible to all children than in large communities (Campbell et al., 2019). This aspect could also help to explain that in the studied sample (from a medium community) RAE was only detected in two FMS. Furthermore, in Spain, it is common for children to go to a school close to their family residence, so it is likely that the participants lived mainly in the same neighborhood where their school was located. That is why it is likely that their environmental and social opportunities to develop their motor skills outside of school were very similar. Also, belonging to the same socioeconomic class would contribute to the absence of differences in their opportunities and social experiences and, therefore, to the almost absence of RAE on their FMS.

In short, and in accordance with other authors, our results show that RAE is not an omnipresent phenomenon (Van Rossum, 2006). The information provided is relevant to facilitate a work plan for education practitioners and other professionals, which can result in important pedagogical and/or therapeutic contributions. In this sense, given the practically absence of differences between our participants born in the first and second semester of the same year, the school organization could be considered adequate based on current criteria (in Spain, natural year of birth), without the need for to propose another criterion for grouping students. However, these results should be viewed with caution and cannot be generalized to other samples given the limitations of this study (which are discussed later).

Referring to interaction effects, significant interaction between level of working memory and gender was found for long jump and total locomotor skills scores. In both cases being a girl and having a higher working memory was associated with obtaining higher scores, while girls with lower working memory had the lowest scores. The results referring to the interaction between working memory and relative age show that only squatting was the task affected by the interaction of these variables. Surprisingly, the highest scores on this FMS task were found in younger participants with higher working memory. More research is needed to explain these results, since it would have been expected that the higher scores were obtained in the older participants, not the younger ones with higher working memory.

Results also revealed a significant interaction between inhibition control and gender for vertical jumps and total dynamic balance scores. In both FMS, the girls with the most inhibition control obtained the highest score in each of these two FMS, while the girls with the lower inhibition control obtained the lowest score in both FMS. These same FMS (vertical jumps and total dynamic balance) are affected by interaction between level of inhibition control and relative age. In both FMS, the oldest participants with higher inhibition control obtained the higher score, while the youngest participants with lower inhibition control obtained the lower score. No significant interactions between gender and relative age were found. It is not possible for us to make a detailed comparison of all these results with those of other studies since we are unaware of studies that have jointly analyzed these same variables. We can only cite the work of Latorre-Román et al. (2021) focused on dynamic balance in preschoolers (but not in the other FMS), where, as in our results, no effect of gender × relative age on dynamic balance was found. More research is needed to analyze and clarify the influences of these and other possible variables on FMS, as well as the mechanisms underlying them.

Despite the fact that with the existing research so far it is difficult to draw firm conclusions about the complex and specific influences of EF, gender and relative age on FMS, the results found in this study have contributed to a greater knowledge about it.

According to the socio-ecological perspective for development, there are numerous variables that affect FMS, not being possible to address all of them in a single study. To our knowledge, this is the first study to address the independent and interactive effects of EF, gender and relative age on FMS in children of such a young age. The results obtained —as has already been indicated in previous paragraphs— provide information of interest both at a theoretical and practical level, so they should be attended by researchers and professionals who work with children, as well as politicians and other stakeholders.

The results obtained in this study contribute to increase the corpus of literature regarding the link between cognition and motor skills, and more precisely, between EF and FMS in kindergarteners. Given that the period from 3- to 6-years is one of very rapid change, both in EF and FMS, it is an important time for examining the relationship between both skills. However, it is a topic so far scarcely studied in such a young age group (Houwen et al., 2017; Hocking et al., 2020), perhaps, among other issues, due to the difficulty involved in research with minors (Dubois et al., 2021). Our study has contributed to diminish this gap. It should also be noted that, although there are neuroimaging, clinical and developmental data in the literature that show that the relationships between EF and FMS are bidirectional (McClelland and Cameron, 2019), until now the investigations have essentially focused on a direction of these relationships (motor skills influences on EF), forgetting the other (EF influences on motor skills) (Musculus et al., 2021; Veraksa et al., 2021). Our study has contributed to reduce this gap by addressing this last direction in the relationships between EF and FMS.

This study also supposes a comprehensive approach on preschoolers’ FMS. Addressing the entire main types of FMS in the same study and assessing each one of them in two motor tasks, and not only in one like most existing research, are two highlights of our study. This has allowed overcoming the limitations of other published studies and responding to the demands of the literature (Van Der Veer et al., 2020; Veraksa et al., 2021). In addition, in our analyses we have considered 13 FMS scores as dependent variables. This also implies a broader perspective of the FMS than usually found in the literature. The in-depth and detailed perspective on FMS adopted in this study has been possible not only because of the number and variety of FMS tasks analyzed but also because of the systematic observation has been used, this aspect being another of the elements to be highlighted in this study.

Spanish educational regulations order that systematic and direct observation is the procedure to be used to assess the development and learning of students in the Early Childhood Education and Care (Spanish Ministry of Education and Science, 2007). Therefore, this study, and in particular the observation instrument used, can be very useful for the daily practice of teachers (more specifically, for the evaluation of the FMS of their students, an aspect that is part of the curricular content of this educational stage). Other free materials and resources elaborated for this research (such as the designed motor tasks) can also be useful for the Early Childhood Education and Care teacher (but also for the sport monitors and trainers) to enhance the FMS of the kids. This is especially relevant given that there are various authors that evidence that teachers, despite recognizing the importance of motor skills in the preschool stage, spend less time learning FMS than other skills because they do not feel sufficiently prepared to address their assessment and teaching (Padial-Ruz et al., 2019; García-Marín and Fernández-López, 2020). There are many teachers who manifest a lack of training in this regard as well as a lack of tools and resources in this area (Aadland et al., 2020; Lawson et al., 2021). This makes increasing the motor training and competence level in professional staff in the preschool sector has become an international challenge (Aadland et al., 2020). As has already been noted, this study provides some elements to achieve it.

In addition, the observation instrument used entail the FMS assessment from both a process and product perspective, a characteristic that is lacking in most of the motor skills assessment instruments used in the literature (Lopes et al., 2021). Furthermore, taking into account that during the childhood the FMS development process is more important than the result of the skill (Palmer et al., 2021), in our observation instrument the process approach predominates over the product. This allows a comprehensive understanding of the strategies employed by children to execute the task and therefore, makes it possible to offer feedback to the child about its execution process, which in turn facilitates its motor learning.

Other aspects to highlight in our study are that (1) it was carried out in the participants’ own school context without altering or modifying it; (2) motor tasks used were designed according to the content of the Early Childhood Education and Care curriculum and taking into account the physical and organizational characteristics of the school and the usual motor sessions that take place in it; (3) these motor tasks were also playful tasks, since play is an essential pedagogical methodology in this educational stage; (4) in the motor sessions analyzed, the teachers used their conventional motor teaching strategies, and (5) direct and systematic observation, which is the methodology required by the educational regulation for assessing children was used to assess the spontaneous behavior of children referring to their FMS. These aspects are in line with ecological approaches claimed by the literature regarding the assessment and intervention of motor skills in similar conditions to real life, in order to improve ecological validity of the research (Woods et al., 2020; Latorre-Román et al., 2021). This helps to establish bridges between research and practice.

On the other hand, it should be mentioned that the use of other free instruments to carry out this study (even though they are not self-elaborated, such as the CHEXI questionnaire and the Lince software) and disseminating/facilitating their access can also help to advance and improve educational practice and research. There are many occasions in which teachers and researchers barely have the budget to carry out our work, even more so taking into account the economic crisis in which we find ourselves worldwide.

Despite the highlights and relevant contributions of this study, these should be considered with some caution given that this study has some limitations. The first and foremost is the use of questionnaires, especially the use of a questionnaire to evaluate children’s EF. It is true that the literature indicates that (1) teacher reports are a useful and attractive option for receiving information about the development of the child given they are time and cost effective, and easy to implement (van Tetering and Jolles, 2017). In fact, this method (rating scales completed by teachers) is one of the most common method used to evaluate child EF (Tamm and Peugh, 2019); (2) Teachers are good informants when filling out questionnaires referring to the EF of their students, since their experience allows them to compare each child with others their age (van Tetering and Jolles, 2017). For all these reasons, it can be said that teacher ratings of EF are good measures of child development (van Tetering and Jolles, 2017; McClelland and Cameron, 2019). However, and despite these positive aspects highlighted in the literature on teacher ratings of EF, the known limitations associated with the use of questionnaires cannot be forgotten, such as assessor variance, also known as rater bias (e.g., the halo effect, central tendency bias, leniency bias) (Waterman et al., 2012). In order to overcome these drawbacks, in the future it would be interesting to complement teacher-rated EF with measures obtained through systematic observation referring to children’s performance in EF tasks in their own habitual context. This implies a more time intensive cost, but in return, systematic observation allows obtaining more and in-depth information, and its objectivity is also greater. Thus, given that both EF observing measures and EF rating measures clearly have their pros and cons (Van Der Veer et al., 2020), their complementary use constitutes an ideal option. Furthermore, this complementarity would be especially interesting insofar as some researchers have suggested that measures assessing EF across informants or by systematic observation tap into different aspects of EF (Toplak et al., 2013; Miranda et al., 2015; Ten Eycke and Dewey, 2016; O’Meagher et al., 2019; Tamm and Peugh, 2019; Van Der Veer et al., 2020). In this sense, in the future it would be interesting to analyze whether, and how, the influence of EF on FMS differs depending on the type of measure adopted to assess EF: indirect (by the teacher’s rating) or direct (observation of child’s behavior) (Ten Eycke and Dewey, 2016). This would contribute to a holistic understanding of the EF-FMS relationships.

Another limitation of this study, as in most of the studies conducted on early childhood (Zeng et al., 2017; Hall et al., 2019), is the size of the sample, which also affects when the gender variable is taken into account to analyze the differences based on it. Furthermore, the sample was not randomly selected and belonged to a single kindergarten. These aspects prevent generalizing the results to all Spanish kindergarteners. Although it is a true challenge (even more so with the current situation caused by the COVID-19 pandemic that restricts entry to educational centers for outsiders, such as researchers), in the future it would be advisable to increase the size of the sample including schoolchildren from different types of centers and located in different cities, being also selected at random. This would allow increasing the power of the analyzes and more transferable statements. However, it cannot be forgotten that expanding the sample could pose a difficulty to be able to assess the FMS through observation, as it has been done in this work. This is so given that systematic observation constitutes an intensive (and not extensive) methodology; in other words, its purpose is to study a small sample but to do it intensively, obtaining more in-depth information (Anguera, 2003). Nonetheless, applying the generalizability theory would allow us to know to what extent the sample size could be increased to generalize the results obtained, while maintaining a satisfactory cost/benefit (Blanco-Villaseñor and Escolano-Pérez, 2017).

On the other hand, it should be noted that although it was tried to create new and challenging motor tasks for children, it could be that this novelty and challenging was not achieved for all children or was not achieved in all designed tasks. Thus, (1) although the teachers affirmed the novelty of the tasks designed for the children in that they had not been worked on in the motor sessions, and (2) only a single trial was permitted for each task, thus avoiding the familiarity and learning that the repeated practice of a task implies, all this does not allow to rule out the possibility that the participants, or some of them, had a certain degree of familiarity with some tasks due to their motor experiences outside the school. Despite we have previously exposed different aspects that suggest that motor experiences outside the classroom did not differ between the participants, some measure should have been assessed to ensure this. Future studies could ask parents about the type of leisure and extracurricular physical-sports activities that their children carry out, as well as the time they dedicate to them. Analyzing these issues in depth and their influence on FMS would be an interesting aspect to address in future research. Although the literature indicates, in general terms, that the practice of extracurricular physical activities favors FMS (Kokštejn et al., 2017), when the results obtained in different studies are analyzed in detail, it is found that not all FMS are benefited by this type of activity. Thus, for example, there are studies that show that in children as young as those that make up this sample, and also Spanish, only some motor components were benefited by these extracurricular activities (Gil-Madrona et al., 2021; Honrubia-Montesinos et al., 2021). Further research is required to clarify what characteristics should have motor extracurricular experiences to enhance the different FMS.

It should also be mentioned as a limitation of this work the fact that it is a punctual study, limitation frequently present in studies focused on early childhood (Zeng et al., 2017; Hall et al., 2019), and therefore the impossibility of establishing causal relationships. Furthermore, longitudinal studies would be necessary to analyze what changes occur in the EF-motor skills relationships as children reach other stages of life.

Likewise, should be noted as a limitation that some possible moderating or confounding variables, such as fitness level of the participants, parenting practices, different characteristics of the teachers such as their personality, experience, etc., in addition to the aforementioned practice of sports extracurricular activities carried out by the children and time dedicated to them (Elferink-Gemser et al., 2018; García-Marín and Fernández-López, 2020; Holfelder et al., 2020), etc., were not contemplated. Thereby, it remains open to future studies.

It would also be interesting in future studies to consider the order and distribution in which the motor tasks were performed. (In this study, as already explained, this question was determined by organizational aspects of the natural context in which the study was carried out). The literature indicates that when motor tasks are learned, it is inevitable that there are relationships between them, either because they are being learned simultaneously, or because a task learned earlier can influence (both positively and negatively) another learned later.

It would be interesting to extend this study to children with disorders in which EF, motor skills, or both are affected. It would allow us a more in-depth knowledge about how EF, gender and age relative influences on motor skills vary in typical and atypical development.

## Conclusion

FMS are a complex area of children development. Our results show that some FMS are specifically affected by independent and interactive effects of teacher-rated EF, gender and relative age. This valuable information must be taken into to design and implement instructional and intervention strategies, as well as educational and sport policy changes, especially in early childhood when FMS are more malleable.

## Data Availability Statement

The raw data supporting the conclusions of this article will be made available by the authors, without undue reservation.

## Ethics Statement

The study was part of a broader research project endorsed by the Research Unit of the university to which the first author belongs. The research was also approved by the school management team. In accordance with Organic Law 15/1999 of December on the Protection of Personal Data (1999, Official State Gazette no. 298, of December 14), all parents of the participants signed the informed consent authorizing their children’s participation in the study and the recording of the children. Furthermore, and following the guidelines of the aforementioned law, observers signed a confidentiality agreement. No special ethical approval was required for this research since the Spanish public education system and national regulations do not require such approval.

## Author Contributions

EE-P was involved in conceptual and methodological structure, literature review, collecting data, systematic observation, manuscript drafting, and discussion. CS-L was involved in methodological structure and data analysis. MH-N was involved in data collection and systematic observation. All authors contributed to revising the manuscript and provided final approval of the version to be published.

## Conflict of Interest

The authors declare that the research was conducted in the absence of any commercial or financial relationships that could be construed as a potential conflict of interest.

## Publisher’s Note

All claims expressed in this article are solely those of the authors and do not necessarily represent those of their affiliated organizations, or those of the publisher, the editors and the reviewers. Any product that may be evaluated in this article, or claim that may be made by its manufacturer, is not guaranteed or endorsed by the publisher.
