# Supplementary material for: Teacher-Rated Executive Functions, Gender and Relative Age: Independent and Interactive Effects on Observed Fundamental Motor Skills in Kindergarteners
Source: Front Psychol. 2022 Feb 22;13:848525. doi: 10.3389/fpsyg.2022.848525 (PMC8904136; doi:10.3389/fpsyg.2022.848525)
Supplement: Supplementary file 1 [file Data_Sheet_1.PDF]

Criterion	*Criterion description*	*Category*	*Category description*	*Category code*
Motor task	Activity performed by the participant	Hopping with one leg	Activity in which the participant should hop on one leg down a line, stepping on it and without crossing it.	Apc
		Long jump	Activity in which the participant, situated on a specific point, jumps forward with both feet together, propelling him/herself with his/her arms. The participant should land on his/her feet, not touching the ground with his/her hands.	Sl
		Throwing a ball	Activity in which the participant should throw a tennis ball horizontally so that it passes through a hoop that is situated at a distance of 1.5 meters away from them.	Lh
		Catching a ball	Activity in which the participant should catch a ball that is thrown by an adult from a distance of 1.5 meters.	Rp
		Squatting	Activity in which the participant, with eyes closed, should remain in a squatting position over the balls of his/her feet, which should be separated by approximately 30 cm, with his/her body bent and arms extended horizontally to the sides (that is, extended in the form of a cross).	C
		Standing on one leg	Activity in which the participant, with eyes closed, should remain standing on one leg, on the same point, first standing on one leg and then changing to the other.	Ppc
		Walking heel-to-toe	Activity in which the participant should walk down a line with his/her heel next to the toes of the other foot (there should be no space left on the ground between the two feet).	Apt
		Vertical jumps	Activity in which the participant, situated on a point that is the center of a square measuring 25 cm per side, and looking forward, should jump up and down ten times in a row, landing on the same point where he/she began.	Ss
Side of a square	Side of the square on which the participant carrying out the activity is situated. This applies in the following tasks: *Hopping with one leg* and *Walking heel-to-toe*.	Side 1	The participant is carrying out the activity on side 1 of the square.	L1
		Side 2	The participant is carrying out the activity on side 2 of the square.	L2
		Side 3	The participant is carrying out the activity on side 3 of the square.	L3
		Side 4	The participant is carrying out the activity on side 4 of the square.	L4
Trajectory	Route or means adopted by the participant´s movement when carrying out the activity; that is, the line described by the participant when he/she moves through the space when carrying out the activity.<br>It applies to the following tasks: *Hopping with one leg* and *Walking heel-to-toe*.	Rectilinear	The participant's movement adopts a straight line. Specifically, this implies that when landing from the jumps (if carrying out the *Hopping with one leg* task*)* or in his/her steps (if carrying out the *Walking heel-to-toe* task), the participant steps on the central line (blue) with the entire foot or part of it.	TR
		Oscillating	The participant's movement is fluctuating, so that in at least one of the landings from their jumps (if carrying out the *Hopping with one leg* task) or in one of their steps (if carrying out the *Walking heel-to-toe* task), the participant does not cross the central line (blue) but instead, steps either to the right (space marked out by the blue stripe and the red one) or to the left (space marked by the blue stripe and green one). That is, in at least one of the landings from their jumps, or in one of their steps, the participant does not step on the central blue line, but on the space that is marked out by the two lateral lines (red and green).	TO
		Erratic	The participant's movement is incorrect, so that, in at least one of the landings from their jumps (if carrying out the *Hopping with one leg* task) or in one of their steps (if carrying out the *Walking heel-to-toe* task), the participant does not step on the central line (blue) or within the space marked out by the two lateral lines (red and green). He/she rather, steps outside this space. That is, the participant steps either very far to their right (beyond the lateral red line) or very far to their left (beyond the lateral green line).	TE
Cadence	Regularity of the series of movements carried out by the participant. It applies in the following tasks: *Hopping with one leg* and *Walking heel-to-toe*.	Rhythmic	The movement is carried out at a constant speed, without interruptions, stops or changes in speed.	CR
		Arrhythmic	The movement is carried out at a varying speed, with interruptions, stops and/or changes in speed.	CA
Extremity	Upper or lower limb (depending on the task at hand) with which the participant carries out the movement. It applies to the following tasks: *Hopping with one leg,*	Right	The (upper or lower) right limb is used by the participant to carry out the movement.	ED
		Left	The (upper or lower) left limb is used by the participant to carry out the movement.	EIz

	Throwing a ball and *Standing on one leg*.			
Arm position	Posture in which the arms are positioned while the participant performs the action. It applies to the following tasks: *Hopping with one leg, Squatting, Long jump* (to each of the jump phases)*, and Throwing a ball.*	Backwards	The arms are placed behind the participant's shoulders while he/she performs the action (which may be a throw from the back to the front or another action with the arm).	PbA
		Forwards	The arms are placed in front of the participant's shoulders while he/she performs the action, without having been, at any time, behind them during the course of the action.	PbD
		Across the body	The arms are extended outwards, following the directionality of the participant's body while he/she performs the action.	PbC
		In the form of a cross with arms extended	Upon performing the activity, the participant keeps his/her arms extended horizontally in the form of a cross.	PbCzE
		In the form of a cross with arms bent	Upon performing the activity, the participant keeps his/her arms horizontally bent in the form of a cross.	PbCzFl
		Others	Upon performing the activity, the participant keeps his/her arms in a position that differs from those mentioned above.	PbO
Jump phase	One of the three phases that may be distinguished in the jump, that is, one of the three moments that can be distinguished in the movement produced by the action of one or both legs, through which the participant's body leaves the ground as a support surface, later returning to it. This applies to the *Long jump* task.	Impulse	Jump phase where the trajectory to be followed by the participant is determined. It requires a bending, followed by a quick extension of the lower trunk.	FsIm
		Flight	Jump phase in which the participant loses contact with the surface. It implies a horizontal displacement across the air.	FsV
		Landing	Jump phase in which the participant comes into contact with the ground once again. In this phase, the accumulated energy is absorbed or transformed.	FsR
Leg position	Posture adopted by the participant's legs when carrying out the following tasks: *Long jump* (in each of the phases of the jump) *and Catching a ball.*	Knees bent	While performing the activity, the participant keeps his/her knees bent, so that his/her abductor muscles and calves are close together.	PRfl
		Knees not bent	While performing the activity, the participant keeps his/her knees extended, without bending them.	PRsfl
Distance to the ground	Space between the ground and the participant's feet. This applies to the following tasks: *Long jump, Squatting* and *Vertical jumps*. In the *Long jump* task, it is applicable to each of the jump phases.	Feet on the floor	The balls of the participant's feet are kept fully on the ground. (In the *Vertical jumps* task, this only applies to the landing of the jump phase).	DsPs
		Heels lifted	Only the balls of the participant's feet touch the ground, with their heels being lifted up from the ground.	DsTe
		Feet in the air	The participant's feet are in the air, not touching the ground with any part of them. This category is not applicable to the *Squatting* task.	DsPa
Centimeters	Space, distance measured in cm from the point where the propelling takes place, as done by the participant when jumping, to the point where the landing of said jump occurs. This only applies to the *Long jump* task.	Quartile 1 distance	The participant has jumped a distance that is situated between 0 and 25% from the lowest distances jumped by the study participants.	C1
		Quartile 2 distance	The participant has jumped a distance that is situated between 26% and 50% from the lowest distances jumped by the study participants.	C2
		Quartile 3 distance	The participant has jumped a distance that is situated between 51% and 75% from the greatest distances jumped by the study participants.	C3
		Quartile 4 distance	The participant has jumped a distance that is situated between 76% and 100% from the greatest distances jumped by the study participants.	C4
Base of support	Space, distance between the participant's feet. This applies to the following tasks: *Long jump* (to each of the jump phases), *Vertical jumps* (to the landing of the jump), *Throwing a ball.*	Feet together	The participant's feet touch one another.	BsPj
		Feet separated	The participant's feet are separated by a distance of less than 15 cm.	BsPs
		Feet widely separated	The participant's feet are separated by a distance of more than 15 cm.	BsPms
Type of landing	Means in which the landing of the jump takes place in the *Long jump* task. This only applies to this task.	Without bouncing	During the landing phase of the jump in the *Long jump* task, the participant keeps his/her feet immobile with respect to the first position in which he/she landed. It is considered a correct jump.	TcSr
		With a bounce	During the landing phase of the jump in the *Long jump* task, the participant moves his/her feet with respect to the first position in which he/she landed. It is considered an incorrect jump.	TcCr
Precision of the	In the *Vertical jumps* task, adjustment between the	The 2 feet within the	During the landing phase of the jump, the participant lands with the 2 feet within the square, without	Ps2Dt

jump	position of the feet in the jump landing phase and the square that is painted on the ground (participant location at the start of the task). That is, this category assesses if the participant, during each jump, lands in the same area where he/she began the task.	square	stepping on any of its lines.	
		At least one foot steps on a line of the square	During the landing phase of the jump, the participant lands stepping on at least one of the lines of the square.	Ps1Dt
		Outside of the square	During the landing phase of the jump, the participant lands with at least one foot completely outside of the square.	PsF
Trunk position	Position adopted by the torso. It applies to the following tasks: *Squatting*, *Throwing a ball* and *Catching a ball*.	Upright	The participant's torso is positioned vertically with respect to the ground, that is, their torso is at a 90º angle to the ground	PtE
		Inclined	The participant's torso is not vertical with respect to the ground, that is, the participant's torso forms an acute or obtuse angle with the ground.	PtI
Time	Seconds in which the participant carries out the activity. It applies to the following tasks: *Squatting* and *Standing on one leg.*	Quartile 1 time	The time that the participant spends performing the activity falls within quartile 1 of the times used by all of the participants.	T1
		Quartile 2 time	The time that the participant spends performing the activity falls within quartile 2 of the times used by all of the participants.	T2
		Quartile 3 time	The time that the participant spends performing the activity falls within quartile 3 of the times used by all of the participants.	T3
		Quartile 4 time	The time that the participant spends performing the activity falls within quartile 4 of the times used by all of the participants.	T4
Direction of lifted leg	Position of the leg that is not standing on the ground. This only applies to the *Standing on one leg* task.	Backwards	The leg that is in the air is positioned behind the shoulders (regardless of the angle formed by the bending of the knee).	DplA
		Laterally	The participant lifts the leg in the air to one side or another (right or left), in the direction of the shoulder (regardless of the angle formed by the bending of the knee).	DplL
		Other	The participant puts the lifted leg in any position other than the previous ones.	DpñO
Angle of the lifted leg	Angle of the leg that is not standing on the ground. This only applies to the *Standing on one leg* task.	Acute angle	The leg that is not standing on the ground is bent at an angle of less than 90º.	AplA
		Right angle	The leg that is not standing on the ground is bent at an angle of 90º.	AplR
		Obtuse angle	The leg that is not standing on the ground is bent at an angle of over 90º.	AplO
Way of catching the ball	Way in which the participant catches the ball that has been thrown, using or not using his/her body to help. This only applies to the *Catching a ball* task.	With both hands	The participant catches the ball with both arms and hands held forward and separated from his/her body, holding it between his/her hands.	Rp2m
		Supporting on body	The participant uses his/her body to catch the ball, supporting the ball with their body.	RAac
		Not catching	The participant does not catch the ball so that it falls to the ground, even if it was previously touched with the hands or another body part, or even when it was previously caught for a brief period of time. (That is, this category is coded regardless of whether the participant momentarily trapped the ball but it immediately fell out of his/her hands).	RpNr
Hand position	Posture adopted by the participant's hands when catching the ball. This only applies to the *Catching a ball* task.	Together	The participant catches the ball with both hands touching each other, with their palms facing upward.	PminJ
		Separate	The participant catches the ball with his/her hands separated, situated one on each side of the ball.	PmS
Height of the catch	Vertical distance at which the ball is recovered with respect to the ground. This only applies to the *Catching a ball* activity.	Chest	The participant catches the ball at chest height.	ArP
		Neck head	The participant catches the ball at face or neck height.	ArCc
		Abdomen	The participant catches the ball at abdomen height.	ArAb
		Thighs	The participant catches the ball at thighs height.	ArM
		Knee	The participant catches the ball at knees height.	ArRd
		Under the knee	The participant catches the ball when it is below his/her knees.	ArDrd
Passing through	It indicates whether or not the ball passes through the hoop. This applies to the *Throwing a ball* task.	Transfer	The ball passes through the hoop.	AtEc
		Touch	The ball touches the hoop but does not pass through it.	AtTn
		Diverted	The ball is diverted and does not pass through or touch the hoop.	AtN
